# L-Carnitine-supplementation in advanced pancreatic cancer (CARPAN) - a randomized multicentre trial

**DOI:** 10.1186/1475-2891-11-52

**Published:** 2012-07-23

**Authors:** Matthias Kraft, Kathleen Kraft, Simone Gärtner, Julia Mayerle, Peter Simon, Eckhard Weber, Kerstin Schütte, Jens Stieler, Heide Koula-Jenik, Peter Holzhauer, Uwe Gröber, Georg Engel, Cornelia Müller, You-Shan Feng, Ali Aghdassi, Claudia Nitsche, Peter Malfertheiner, Maciej Patrzyk, Thomas Kohlmann, Markus M Lerch

**Affiliations:** 1 Department of Medicine A, University Medicine Greifswald, Friedrich Löffler Straße 23a, Greifswald, 17475, Germany; 2 Department for Gastroenterology, Hepatology and Infectious Diseases, Otto von Guericke University Magdeburg, Magdeburg, Germany; 3 Charité Universitaetsmedizin Berlin, Campus Virchow-Clinic, Medical Clinic Hematology/Oncology, Berlin, Germany; 4 Veramed Clinic, Brannenburg, Germany; 5 Academy of Micronutrient Medicine, Essen, Germany; 6 University Pharmacy, University Medicine Greifswald, Greifswald, Germany; 7 Institute of Clinical Chemistry and Laboratory Medicine, University Medicine Greifswald, Greifswald, Germany; 8 Department of Surgery, University Medicine Greifswald, Greifswald, Germany; 9 Institute of Community Medicine, University Medicine of Greifswald, Greifswald, Germany

**Keywords:** Pancreatic adenocarcinoma, L-Carnitine, Quality of life, Survival, Cancer cachexia, Fatique syndrome

## Abstract

**Background:**

Cachexia, a >10% loss of body-weight, is one factor determining the poor prognosis of pancreatic cancer. Deficiency of L-Carnitine has been proposed to cause cancer cachexia.

**Findings:**

We screened 152 and enrolled 72 patients suffering from advanced pancreatic cancer in a prospective, multi-centre, placebo-controlled, randomized and double-blinded trial to receive oral L-Carnitine (4 g) or placebo for 12 weeks. At entry patients reported a mean weight loss of 12 ± 2,5 (SEM) kg. During treatment body-mass-index increased by 3,4 ± 1,4% under L-Carnitine and decreased (−1,5 ± 1,4%) in controls (p < 0,05). Moreover, nutritional status (body cell mass, body fat) and quality-of-life parameters improved under L-Carnitine. There was a trend towards an increased overall survival in the L-Carnitine group (median 519 ± 50 d versus 399 ± 43 d, not significant) and towards a reduced hospital-stay (36 ± 4d versus 41 ± 9d,n.s.).

**Conclusion:**

While these data are preliminary and need confirmation they indicate that patients with pancreatic cancer may have a clinically relevant benefit from the inexpensive and well tolerated oral supplementation of L-Carnitine.

## Background

Adenocarcinoma of the pancreas is highly resistant to chemo- or radiotherapy [1], has a 5-year survival rate of only 4% and ranks as fourth leading cause of cancer death [2-4]. One reason contributing to this high mortality is cancer cachexia, defined as an unintended weight loss of more than 10% in 6 months, which is present in more than 80% of pancreatic cancer patients [5]. Cancer cachexia is also a predictor for reduced quality of life, increased mortality, and poor response to therapy [6-10]. A deficiency of L-Carnitine has been proposed to be an underlying cause of cancer cachexia [11] and tumor associated fatigue [12-14]. Although L-Carnitine can be generated via endogenous conversion from lysine and methionine, 75% of the required levels are provided from food sources. In vitro studies in human tumor cell lines have shown a positive effect of L-Carnitine regarding the inhibition of apoptosis and DNA-damage [15]. On the other hand, L-Carnitine is well known for its potential to modulate the inflammatory response mechanisms, which is known to play the predominant role in the generation of cancer cachexia, especially in pancreatic tumor patients [16]. We therefore conducted a multicentre trial to investigate the role of oral L-Carnitine supplementation on cancer cachexia in pancreatic cancer (CARPAN).

## Methods

**Figure 1:**
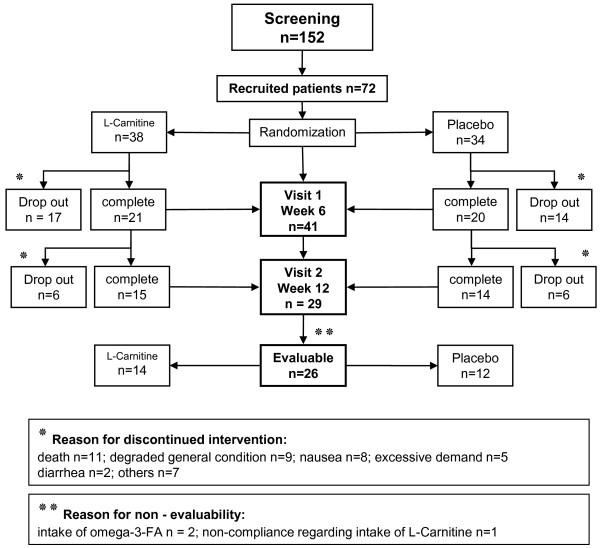
Flow chart of the trial.

Patients from 4 participating tertiary referral centers were considered eligible for inclusion when they had histologically proven, advanced and irresectable adenocarcinoma of the pancreas (UICC Stage IV), had a Karnofsky performance status of >60 and declared their written informed consent to participate. The CARPAN protocol was approved by the ethics committee of Greifswald University (Reg.Nr.IIIUV73/05) and registered at clinical-trials.gov (NCT01330823) and under ISRCTN83465351. Patients were recruited regardless of concomitant or scheduled chemotherapy. Exclusion criteria were liver failure, a second malignancy, treatment with omega-3-fatty acids and the presence of a mental disorder precluding informed consent. From May 2006 until October 2009 a total of 152 patients were screened and 72 enrolled in the study (Figure 1). Reasons for non-enrollment were mostly due to poor performance status or withheld consent. Patients were randomized (sequential series of 4 per block, sealed envelopes, computer generated randomization code) to receive either an oral liquid formulation of L-Carnitine (4 g/d, obtained from Lonza, Basel, CH) or identically formulated placebo with follow up visits at 6 and 12 weeks after entry. Compliance was tested by determining serum L-Carnitine levels by Tandem-Mass-Spectrometry (normal range between 40–60 μmol/l) (ABI 2000, Perkin-Elmer, Turku, SF) [17]. L-Carnitine deficiency is generally believed to occur below 30 μmol/l, albeit data on functional relevance are controversially discussed [18]. At every study visit adverse events and body mass index (BMI) were recorded and bioelectrical impedance analysis (BIA-Nutrigard-M, Darmstadt, Germany) was used to determine body composition [19]. For evaluation of quality of life we used the EORTC-QLQ-C30 questionnaire with a pancreatic cancer specific module PAN26 [20] and for fatigue the Brief Fatigue Inventory (BFI) questionnaire [21]. Survival time in days was calculated from time of diagnosis to death. Sample size calculation was based on previous studies investigating the effect of L-Carnitine on inflammatory markers [22], with TNFα level differences as primary endpoint, and resulted in a recruitment goal of 90 patients (45 per treatment arm) for a statistical power of 90% with an error probability of <5%. After a prescheduled interim analysis for sample size recalculation of 72 blinded datasets showed a wide variation of the standard errors for inflammatory markers a recruitment of 554 patients (277 per group) would have been necessary. Since this goal was unattainable the study was closed after enrolment of 72 patients and the data were unblinded for statistical analysis.

Data are presented as means ± SEM and 95% confidence intervals where appropriate. Statistical analysis for intention-to-treat and per-protocol analysis was done by Student’s *t* test and Pearson's chi-square test for parametric and Mann–Whitney-U-Test for non-parametric analysis. Quality of life data were analyzed using ANOVA. Results were considered significant when p was <0,05.

## Results

Patient characteristics are given in Table 1 and showed no statistical difference between both groups at enrolment.

**Table 1:** Characteristics of the study population (n = 72) at baseline visit of the study (mean ± SEM)

**Parameter**		**L-Carnitine (n = 38)**	**Placebo (n = 34)**
gender	male	20 (52.6%)	23 (67.6%)
	female	18 (47.4%)	11 (32.4%)
mean age		64.4 ± 1.67	64.4 ± 1.65
Karnofsky performance status		76.8 ± 1.87	80.0 ± 2.16
**Nutritional Status**			
normal BMI (kg/m²)		28.0 ± 1.01	30.1 ± 0.84
baseline visit BMI (kg/m²)		24.7 ± 0.65	24.9 ± 0.89
Phase angle (°)		4.4 ± 0.16	4.4 ± 0.17
Weight loss*	present	34 (89.5%)	31 (91.2%)
	absent	4 (10.5%)	3 (8.8%)
meanweight loss (kg)*		11.4 ± 1.28	12.3 ± 1.56
Nutritional support	none	20 (52.6%)	21 (61.8%)
	oral	14 (36.8%)	9 (26.5%)
	Parenteral nutrition	4 (10.5%)	4 (11.7%)
ECM/BCM index **		1.5 ± 0.11	1.4 ± 0
Cell percentage (%)		41.8 ± 1.22	42.70 ± 1.21
chemotherapy (n) 35 (92%) 30 (88%)			
**Laboratory values**			
L-Carnitine level (μmol/l)		25.3 ± 2.29	24. 8 ± 2.11
Albumine (g/l)		33.8 ± 1.09	33.7 ± 1.20
CRP (mg/l)		31.3 ± 6.55	45.5 ± 10.39
Leucocytes (Gpt/l)		8.3 ± 0.83	6.9 ± 0.46
CA 19–9 (U/ml)		14,095 ± 32,572	18,345 ± 35,950

No statistically significant differences were observed between L-Carnitine and placebo group.

*weight loss during the last 6 month.

**ECM/BCM-Index, Extra-Cellular-Mass/Body-Cell-Mass-Index.

**Figure 2:**
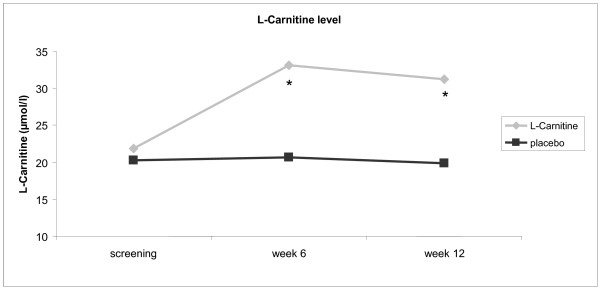
**Reasons for discontinued convention of the study patients.** Reasons for discontinued convention of the study patients.

At entry 88% of patients in the placebo and 92% of patients in the L-Carnitine group received chemotherapy. There was no statistically significant difference between both groups (p < 0,05). 90% of the patients reported a weight loss of >10% during the previous 6 month. This observation is in line with previous reports on cancer cachexia [5]. 26 patients completed the entire follow up period and premature drop-out was due to death (n = 11), deteriorating health (n = 9), nausea (n = 8), excessive demand (n = 5), diarrhea (n = 2) or miscellaneous symptoms (n = 7). Drop out rates and reasons were not different between both treatment arms. Oral supplementation of L-Carnitine substantially increased L-Carnitine serum plasma levels up to 60% of the basic value at week 6 (p < 0,009) in the L-Carnitine group (Figure 2), while a constant decline of L-Carnitine plasma levels was evident during the observation period in the placebo group. This might be related to tumor effects and / or due to concomitant chemotherapy. L-Carnitine supplementation was well tolerated. Side effects did not differ significantly in comparison with placebo group (predominantly nausea, vomiting, and diarrhoea) and, whenever they occurred, may have been caused by concomitant chemotherapy.

**Figure 3:**
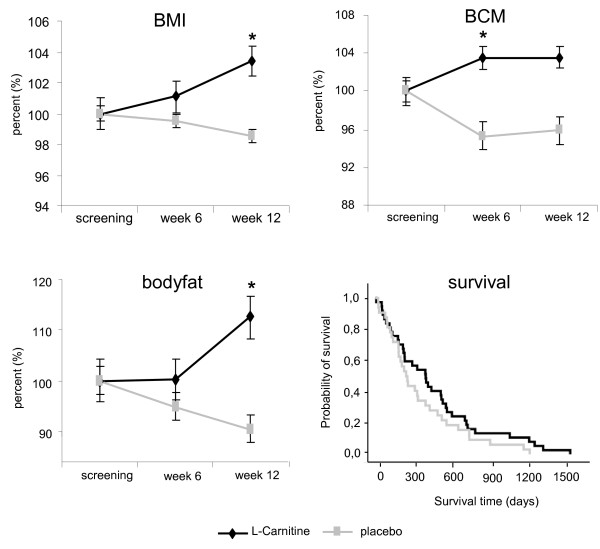
**Relevant nutritional parameters and survival.** Relevant nutritional parameters (means ± SEM) and survival in days in the L-Carnitine treatment arm (black lines) and placebogroup (gray lines). Survival is given in days after diagnosis as Kaplan-Meier curve and body mass index (BMI), body fat, and body cell mass (BCM) aregiven as percent changes under respective treatment over 12 weeks. Asterisks indicate statistically significant differences (p < 0.05).

Patients on L-Carnitine treatment gained weight (BMI increase of 3.4% ± 1.35)whereas patients on placebo did not (BMI reduction of 1.5% ± 1.4, p < 0.018). After 12 weeks of therapy the difference amounted to 4.9% ± 1.9 (Figure 3) between groups. BIA revealed that this improvement was due to increases in body cell mass (BCM, p < 0,013) and body fat (BF, p < 0,041). CRP, albumin, leukocyte count and CA19-9 remained unaffected (data not shown). Regarding quality of life (EORTC-QLQ-C30/PAN26) the only significant changes were improvement in cognitive function (at enrolment 81,0 ± 21,5 in L-Carnitine group, 86,1 ± 17,2 in placebo group; after 6 -weeks L- Carnitine group 0,30 versus −0,13 in the placebo group, p < 0,034), improvement of global health s\tatus (at enrolment 53,6 ± 19,5 in L-Carnitine group, 65,3 ± 17,7 in placebo group; after 12 weeks L- Carnitine group 0,76 versus −0,32 in the placebo group, p < 0,041) and reduction in gastrointestinal symptoms (at enrolment 29,8 ± 32,1 in L-Carnitine group, 19,4 ± 24,5; in the placebo group; after 12 weeks L-Carnitine group −0,35 versus 0,78 in the placebo group; p < 0,033). Differences in fatigue (moderate/severe, >4 on BFI), present in 28,6% of L-Carnitine patients versus 41,7% in the placebogroup,were not statistically significant, nor was the survival benefit (Figure 2, median 519 ± 50d versus 399 ± 43d with placebo), and the reduction in length of hospital stay (36 ± 4d versus 41 ± 9d with placebo).

## Conclusion

Cancer cachexia and malnutrition are associated with an increased risk of surgical complications and higher toxicity levels of chemotherapy. Quality of life and overall survival of colon cancer patients can improve under early nutritional intervention [23]. L-Carnitine is critical for energy generation by mitochondrial ß-oxidation and was found depleted under chemotherapy [24-26]. Its oral supplementation can normalize nutritional L-Carnitine deficiency [27,28] and reduce chemotherapy related side effects [29,30]. We therefore tested whether oral L-Carnitine supplementation has a clinical benefit in patients with advanced pancreatic cancer and found that L-Carnitine can reduce malnutrition, increase bodyweight and improve body composition.

When we planned and designed the study no persuasive data for clinical endpoints existed and we had to base the initial power calculations on inflammatory markers in humans under L-Carnitine treatment. While this study must be regarded as preliminary because inflammatory markers were found to be an unsuitable primary endpoint in our setting, the CARPAN trial provides the basis for a robust sample size calculation: a conclusive study of L-Carnitine benefits ought to enrol 148 patients in each arm to show a survival benefit with 90% power, and would need 157 patients in each arm to demonstrate an improvement in tumor fatigue. The presently available data show a benefit of L-Carnitine supplementation on body weight, body composition and some aspects of quality-of-life, even though it was underpowered to determine the statistical significance of other secondary endpoints. While the loss of significant changes in BCM at week 12 and a rapid increase in body fat between week 6 and 12 was an unexpected finding it might be explained by the underlying progressive tumor disease leading to changes in body composition, irrespective of gain of weight and due to reduced physical activity and progressive sarcopenia. Both are common findings in pancreatic tumors. In this context it is important to note, that the direct influence of L-Carnitine on tumor growth has not been measured in this study and is beyond the scope of this work. Future studies are needed to address this question as only recently L-Carnitine has been reported to modify apoptosis and DNA-damage in tumor cell lines of the brain via CPT-1 C [15].

The CARPAN trial could suggest that the clinical benefit of an inexpensive and very well tolerated oral L-Carnitine supplementation may reach the clinical benefit level previously shown for palliative Gemcitabine chemotherapy [31] in pancreatic cancer patients.

## Abbreviations

BIA, Bioelectrical impedance analysis; BF, Body fat; BFI, Brief fatigue inventory; BMI, Body mass index; BCM, Body cell mass; CPT, Carnitine palmitoyltransferase; CRP, C-reactive protein; ECM, Extra cellular mass; SEM, Standard error of the mean; TNFα, Tumor nekrose faktor α.

## Competing interests

The authors declare that they have no competing interests.

## Authors’ contribution

All authors of the study substantially contributed to conception, design, acquisition and analysis of data and interpretation of the study. KK, MK, MML, UG, and PH designed research, SG, JM, PS, KS, JS, HKJ, AA, CN, PM and MP conducted research, JR, MF, HV and CDH provided essential materials, CM, GE, EW, YSF and TK analysed data, KK, MK and MML wrote paper, KK, MK and MML had primary responsibility for final content. All authors read and approved the final manuscript and disclose any financial or personal relationships.
